# “How would you handle this?” The impact of embedding early patient and public involvement in a biomechanical computational engineering doctoral research project

**DOI:** 10.1186/s40900-025-00694-3

**Published:** 2025-03-18

**Authors:** Tinashe Munyebvu, Gloria Lillywhite, Nehruvita May, Charles Burson-Thomas, Carmel McGrath, Cheryl Metcalf, Martin Browne, Alex Dickinson

**Affiliations:** 1 Bioengineering Science Research Group, Department of Mechanical Engineering, Faculty of Engineering and Physical Sciences, University of Southampton, Southampton, UK; 2 Independent Public Contributor, Southampton, UK; 3 NIHR Health Protection Research Unit in Behavioural Science and Evaluation, University of Bristol, Bristol, UK; 4 The National Institute for Health and Care Research Applied Research Collaboration West University Hospitals Bristol and Weston NHS Foundation Trust, Bristol, UK; 5 Faculty of Health and Applied Sciences, School of Health and Social Wellbeing, University of West England, Bristol, UK; 6 School of Healthcare Enterprise & Innovation, Faculty of Medicine, University of Southampton, Southampton, UK

**Keywords:** Public involvement, Public engagement, Engineering, Biomechanical engineering, Doctoral research, Computational modelling, Hand osteoarthritis

## Abstract

**Background:**

Engineering is often described as a technology-driven field. However, whilst frameworks exist to engage with stakeholders, patient and public involvement (PPI) is not often undertaken in projects that have a quantitative methodology, such as engineering. This can have an impact on research quality, relevance, accessibility and experience. This is especially significant in a biomechanical engineering context where the end-user is often a person with an experience or living with a condition that the researcher does not have.

**Aim:**

This paper aims to provide a commentary on the first steps taken to embed PPI into a biomechanical engineering doctoral research project, and the outcomes and learnings that have come from this experience.

**Methods:**

Three members of the public living with hand osteoarthritis (OA) were involved in the early-stage PPI consultations. These sessions aimed to openly discuss the hand OA lived-experience, current treatments and considerations for the project.

**Results and Discussion:**

Early-stage PPI allowed a deeper understanding of the hand OA lived experience and prompted further PPI activity within the biomechanical engineering research project. Subsequently, a long-term partnership with public contributors was established, shifting the project’s focus from purely developing a computational model to addressing three PPI-identified priorities: (1) patient variability, (2) joint instability, and (3) raising hand OA awareness, using both computational modelling and public engagement methods. Though the number of contributors was small, it allowed for meaningful and long-lasting partnerships to be developed. Based on the learnings from this approach, eight recommendations were developed for researchers seeking guidance on integrating PPI in similar research. These include leveraging the power of storytelling, introducing PPI into the research as early as possible, investing in training and planning, establishing a meaningful partnership with members of the public, understanding the commitment, maintaining flexibility, providing consistent feedback and diversifying research efforts.

**Conclusion:**

This project has demonstrated PPI can inspire ideas and guide critical thinking and technical workflow, uncovering solutions that might not emerge without collaboration. Although the evidence-base is limited, we advocate that PPI has a place in quantitative-heavy research fields such as engineering, especially biomechanical engineering where people are often the end-users of research outcomes.

**Supplementary Information:**

The online version contains supplementary material available at 10.1186/s40900-025-00694-3.

## Abstract (plain language summary)

Patient and public involvement (PPI) is not often undertaken in engineering-based research projects. This commentary presents the process and impact of integrating PPI into a biomechanical engineering doctoral research project about the hand joints. Members of the public living with hand osteoarthritis (OA) were consulted at the start of the project to identify research priorities from a lived-experience perspective. Following these consultations, an active and long-term partnership with these public contributors was established. Patient variability, joint instability and raising OA awareness were identified as research priorities to address using computational modelling and public engagement methods. As a result, this project evolved from a technology-driven biomechanical engineering project to one driven by PPI. We present a set of learnings from this experience that highlight practical priorities for researchers seeking to include PPI in their research.

**Supplementary Information:**

The online version contains supplementary material available at 10.1186/s40900-025-00694-3.

## Introduction

### The social responsibility of engineers

According to the Engineering Council, engineering activity *“can have a significant societal impact and engineers must operate in a responsible and ethical manner, recognise the importance of diversity, and help ensure that the benefits of innovation and progress are shared equitably and do not compromise the natural environment or deplete natural resources to the detriment of future generations.”* [1]. Engineering, as a culture rather than a practice, has sometimes been viewed as a field that is technology-focused, socially isolated, and with aims that do not always align with community objectives [2–4]. For instance, in their 2014 paper, Cech describes a *“culture of disengagement”* in engineering education. They define this as *“a constellation of beliefs, meanings and practices that frame the way profession members conceptualize their professional responsibility to the public.”* [4]. Cech attributes this culture of disengagement to be most evident in the process of defining the problem (i.e. the first stage of the research cycle which involves identification and prioritisation), where they directly state, *“engineers decide what considerations are integral to their design responsibilities for a particular technological puzzle and what concerns they can bracket”*. According to Cech, this approach can lead to an exclusion of non-technical stakeholders and public welfare considerations. In the subsequent decade there has been a push in engineering education to encourage better community engagement and train engineers who possess and value a diverse range of technical and non-technical skills [5–7]. The term ‘holistic engineer’ has been widely adopted to represent engineers who possess knowledge and skills beyond technical expertise. These professionals have non-technical skills that come from an awareness of their ethical and professional responsibility and the societal impact of engineering. This is emphasised by Canney and Bielefeldt who state that *“recognizing the many non-technical dimensions of engineering projects is central to our view of social responsibility because it focuses on identifying the needs of others and working with all affected parties to find appropriate solutions.”* [7].

The Professional Social Responsibility Development Model (PSRDM) [8] explores the attitudes of individual engineers toward professional responsibility and their role in addressing societal issues. To achieve this, engineers must develop both personal social awareness and professional skills, along with an understanding of their combined strength. The National Institute for Health and Care Research (NIHR) defines Patient and Public Involvement (PPI) as research carried out with or by members of the public rather than to, about or for them [9–11]. Thus, PPI and its associated democratic principles may be an enabler of a PSRDM development model as it advocates a public-researcher partnership to address the needs of those who may benefit or be otherwise impacted by the research [12, 13].

### The scope for PPI in biomechanical engineering

Biomechanical engineering is an interdisciplinary field that benefits from collaboration with non-engineers such as but not limited, clinicians, surgeons, and policymakers. However, it remains largely technology-driven. PPI in research ensures that studies focus on outcomes important to the public. Literature shows that PPI in health research can enhance design, quality, relevance, accessibility, and experience [14–17]. There exists a wide range of health research studies that have embedded PPI in their work, including but are not limited to cancer [18, 19], mental health [20] and HIV [21, 22] research. Although PPI has been actively encouraged by engineering research bodies in recent years [1, 23, 24], PPI is rarely implemented or reported in biomechanical engineering projects, particularly those using quantitative methodologies, which may discourage researchers with limited PPI experience from integrating it into their work.

The UK’s National Health Service (NHS) reported limited evidence of PPI impact on quantitative data analysis and attributed this to a lack of involvement rather than reporting, highlighting the scope for more PPI in quantitative-based projects [25]. In their systematic review of published PPI literature in health research published between 1995 and 2009, Boote et al. [26] found more examples of the public being involved in qualitative compared with quantitative empirical research, speculating that researchers may find it easier to involve the public in qualitative rather than quantitative research. However, statisticians, Pfannkuch and Wild, state that context knowledge is needed to do even the most purely technical role effectively [27]. PPI represents a means of addressing the democratic principles of research and recognises that patients/public *“have a personal experience of disease that is not available to most researchers, but that complements researchers’ analytical skills and scientific perspective.”* [28]. This is particularly significant in a biomedical field where the public are the end-users of engineering research outcomes. These are the exact principals that encouraged the inclusion of the lived experience perspective in the biomechanical engineering doctoral research project discussed in this commentary.

When we refer to “engineering” being largely technology-driven, we are referring to engineering in a “research” context rather than a “design” context. Design engineers are relatively conscious of the needs of users and other stakeholders, and this is evidenced through the existence of the Design Council who advocate for frameworks such as the Double Diamond or IDEO, which have been practising human-centred design since their beginning in 1978 [29, 30]. Similarly, areas of research where the engineering is more linked to a kind of ‘designed’ product/device whose use is the person’s/patient’s choice are more likely to use public-centred approaches. Therefore, this paper puts forward an argument for more active PPI in biomechanical engineering research projects whereby the public-centred approach is less established, such as in a computational modelling context.

## Clinical context: project background

Osteoarthritis (OA) is the most common musculoskeletal joint condition, affecting millions of people in the ageing global population [31–35]. OA in the hands is highly prevalent [36, 37]. As well as causing chronic pain, hand OA can have a significant impact on hand function, limiting an individual’s ability to perform everyday tasks.

Computational modelling is often used in biomechanical engineering to assess joint mechanics and degeneration in conditions such as OA [38]. In 2020, a four-year doctoral research project began with the initial aim of leveraging a unique dataset of finger kinematics, computed tomography (CT) and magnetic resonance (MR) imaging of ten consenting participants (5F:5 M, mean age 31yrs, range 27 – 37yrs), who were free from hand or wrist disease or injury (IRAS Ref: 14/LO/1059). The data was collected between 2012 and 2016 at the University of Southampton/Southampton General Hospital [39] and ethical approval was granted for Secondary Data Analysis use (ERGO Ref: 61718). However, at the conception of the research project, it was not decided how the datasets would be used to develop a computational model. Suggestions such as evaluating treatment options and rehabilitation strategies were proposed by members of the interdisciplinary research team. However, a public perspective was lacking, and it was important for us to include voices that represented the OA lived-experience to better understand how these models can be used to support end-users in the future.

The quantitative and largely independent nature of computational modelling especially when compared to clinical studies whereby engaging with the public is a fundamental part of the process, made this particular project an appropriate case study for exploring the impact of PPI in engineering fields where it is less established. Consulting members of the public was initially intended for the project planning stages. However, PPI is often encouraged to be actively incorporated at every possible stage, and the enthusiasm from both the contributors and researchers during the first few meetings about this project evidenced the value of maintaining and deepening this partnership. This commentary is the story of the project’s restructure, whereby after early-stage work with the public contributors, it evolved from a technology-driven biomechanical engineering project to a PPI-driven biomechanical engineering project, which incorporated both computational modelling and public engagement methods to address PPI-identified research priorities.

This first-hand account of PPI in an engineering context is co-authored by the doctoral research student (TAM), two public contributors (GL and NM) and five supporting academic members of staff. The author group worked together for 4 years. Written and verbal consent was obtained to include all information shared in this publication.

## Aim

This commentary aims to describe:

1. how we first implemented public involvement in a biomechanical engineering doctoral research project;
2. the outcomes of the early-stage PPI work and how it informed the project structure; and
3. the overall process and impact of PPI on the research project.

## Methods

This publication was written in accordance with the short-form Guidance for Reporting Involvement of Patients and the Public (GRIPP2) checklist [40]. The following sections are dedicated to outlining the design and implementation of the early-stage PPI consultations which influenced the initial reprioritisation of the PPI elements of the engineering project.

### Terminology used

We use ‘public contributor’ to describe the members of the public on the research team. Throughout this paper, we refer to Patient and Public Involvement (PPI) when discussing the public contributors’ influence on the project’s design, development and delivery. Patient and Public Involvement and Engagement (PPIE) is used to describe activity that encompasses both involvement *and* engagement.

### People involved

Three members of the public were involved within the first six months of the project, with two of the three public contributors continuing their involvement throughout the project’s duration. All three contributors were female, over fifty years old, currently living with either clinically confirmed or suspected OA in their hands. The severity of symptoms vary between the contributors, ranging from mildly to severely impacting their quality of life. They all volunteered to be part of this project and had no previous experience of PPI to this degree. They were recruited through the Saints Foundation—a charitable organisation run by a local football club [41]. Saints Foundation are a ‘social prescriber’ for the NHS. They provide weekly exercise sessions that promote regular exercise and independence within the local community. Reimbursement was offered to all public contributors involved.

### Stages and nature of involvement

The initial consultations during the project design stage were conducted in accordance with the GRIPP2 checklist [40], UK Standards for Involvement [11], and NIHR’s briefing PPI notes for researchers [42]. Ethical approval was not required as persons were acting in an advisory role (Briefing Note 5). However, ethical approval (ERGO62720) was sought for dissemination purposes, allowing consent to capture the discussions and contributors’ lived-experiences in the form of interactive and collaborative notes (Jamboard, Google Inc., California, USA), and share them at conferences, in publications or on social media.

A ‘terms of reference’ (see Supplementary File 1) document was created and distributed to everyone who would be present during the session. This document included information on the purpose, location and format of the meetings, how the meetings would be conducted and data protection considerations. We recognised that there wasn’t much guidance or case-study based evidence of PPI in this field of research and thus, we sought out shadowing opportunities of colleagues undertaking PPI to help gain knowledge in how to run meetings, capture impact and reimburse contributors.

Four one-hour sessions were held online via video conferencing software (Zoom, Zoom Communications, Inc., California, United States of America), due to transmission control rules during the COVID-19 pandemic. Each session was organised around discussion prompts (Table 1). These discussions were made as general as possible to ensure that the conversation remained open to all perspectives to minimise the risk of researcher bias. This was also done to reassure public contributors that there is no ‘right answer’ and allow both the researchers and public contributors to learn from each other in a spontaneous manner. As well as open conversations, discipline-specific language was avoided to ensure that the conversation was accessible to everyone, and feedback would be readily given.

**Table 1:** Discussion Prompts for each consultation session

Session no	Discussion prompt
1	∙ What are your opinions on currently available OA treatments? ∙ What do you think of this computational modelling research project?
2	∙ What movements/actions are most difficult to do living with hand OA? ∙ What considerations should researchers have in mind when developing tools to investigate OA?
3	∙ What activities of daily living are mostly impacted by hand OA? ∙ What techniques do you use to manage your symptoms?
4	∙ What is the public perception of hand OA? ∙ What recommendations would you give to researchers developing treatment?

## Capture or measurement of early-stage consultations

Points discussed verbally during the sessions were recorded in the form of hand-written and interactive electronic notes (Jamboard, Google Inc., California, USA). These notes acted as a written record of the session and were sent via email to attendees. This allowed everyone to access, edit, and delete any information they felt did not represent what was discussed. After the last session, the notes were collected, summarised and reviewed by all parties. The process and impact of this public involvement work on the doctoral project was evaluated using impact logs (see Supplementary File 2) adapted from a People in Health West of England (PHWE) template [43].

We created spaces for reflection to ensure the interpretation of the discussion was accurately documented. For instance, both researchers and public contributors were invited to write blog posts that would be published online to depict their experience during this stage of the project [44].

## Study results and discussion

### Becoming a PPI-driven biomechanical engineering project

During the sessions, public contributors were keen to share their views and expertise. A particularly important aspect of the discussions was the impact of hand OA upon everyday living.

*“[The researchers] … who were running these sessions were quite surprised by some of the things that are affected by arthritis and what we have to do to overcome it. Simple things we take for granted like sewing, writing a letter, opening those childproof caps, or trying to. Even gripping a bread knife or picking something up, when we are in the throes of a flare-up can be nigh on impossible.”*

*– Public contributor talking about the researchers* [44]

**Fig. 1:**
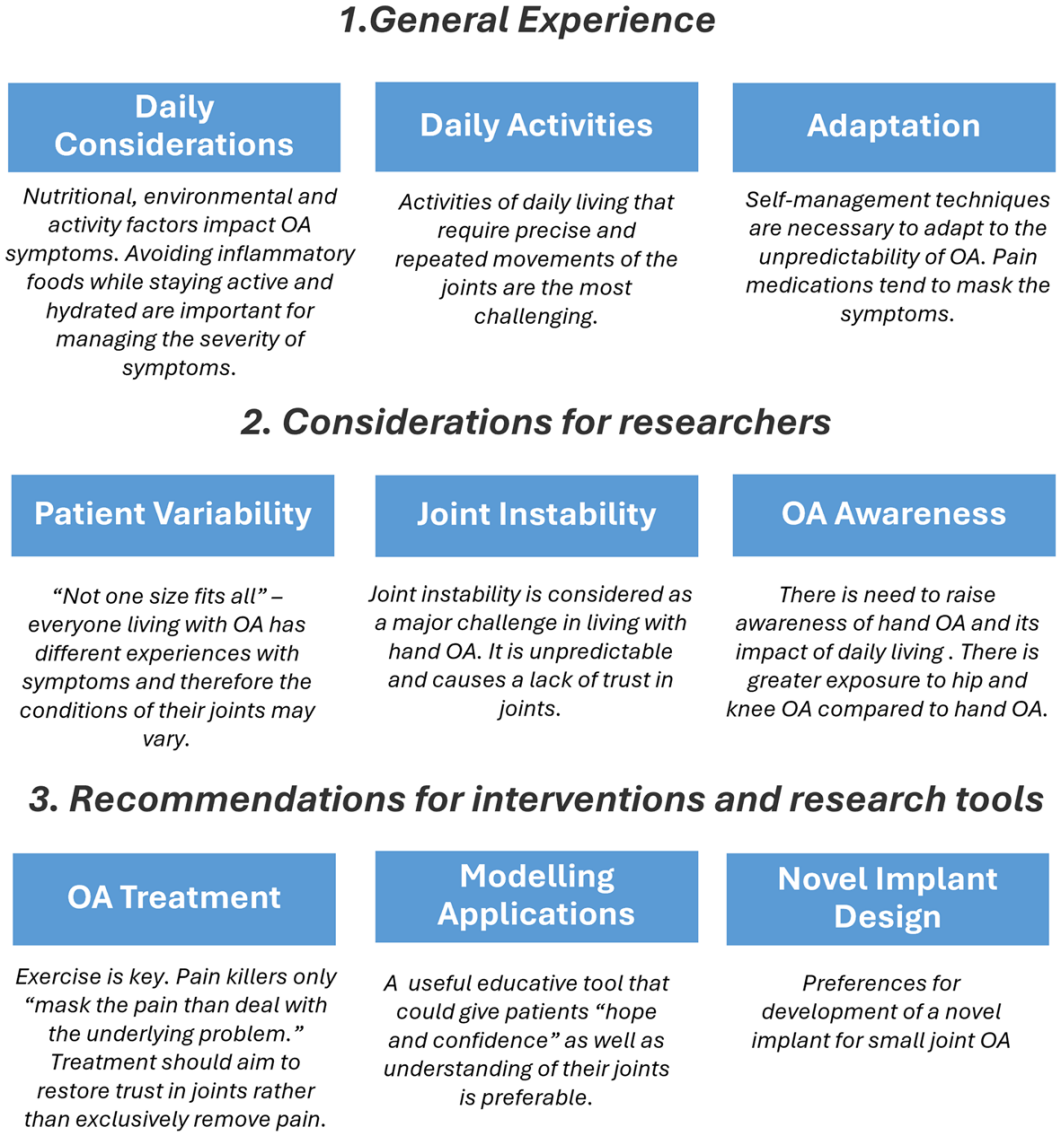
Key hand OA lived-experience factors discussed during early-stage PPI consultations

The discussions about lived-experience ultimately outlined the main factors that the public contributors felt could be considered in biomechanical engineering research. As a team (researchers and public contributors), we explored these factors and self-categorised them into three main groups (Fig. 1):

1. General Experience: Factors that describe daily experience of living with hand OA.
2. Considerations for researchers: Factors that public contributors feel could be more thoroughly considered in biomechanical research about hand OA
3. Recommendations for interventions and research tools: Opinions on research tools and inventions that researchers could also consider.

The discussions with public contributors allowed for an improved governance of the project as a whole. For myself as the researcher (TAM), it widened my understanding of hand OA and its impact on everyday life, opening up the discussions to topics that I had yet to prioritise in my literature search and determination of the research question. Overall, the discussions with the public contributors encouraged me to look beyond the computational methods as the main project output and instead, explore the ways that the project could address the priorities identified by the public contributors.

**Fig. 2:**
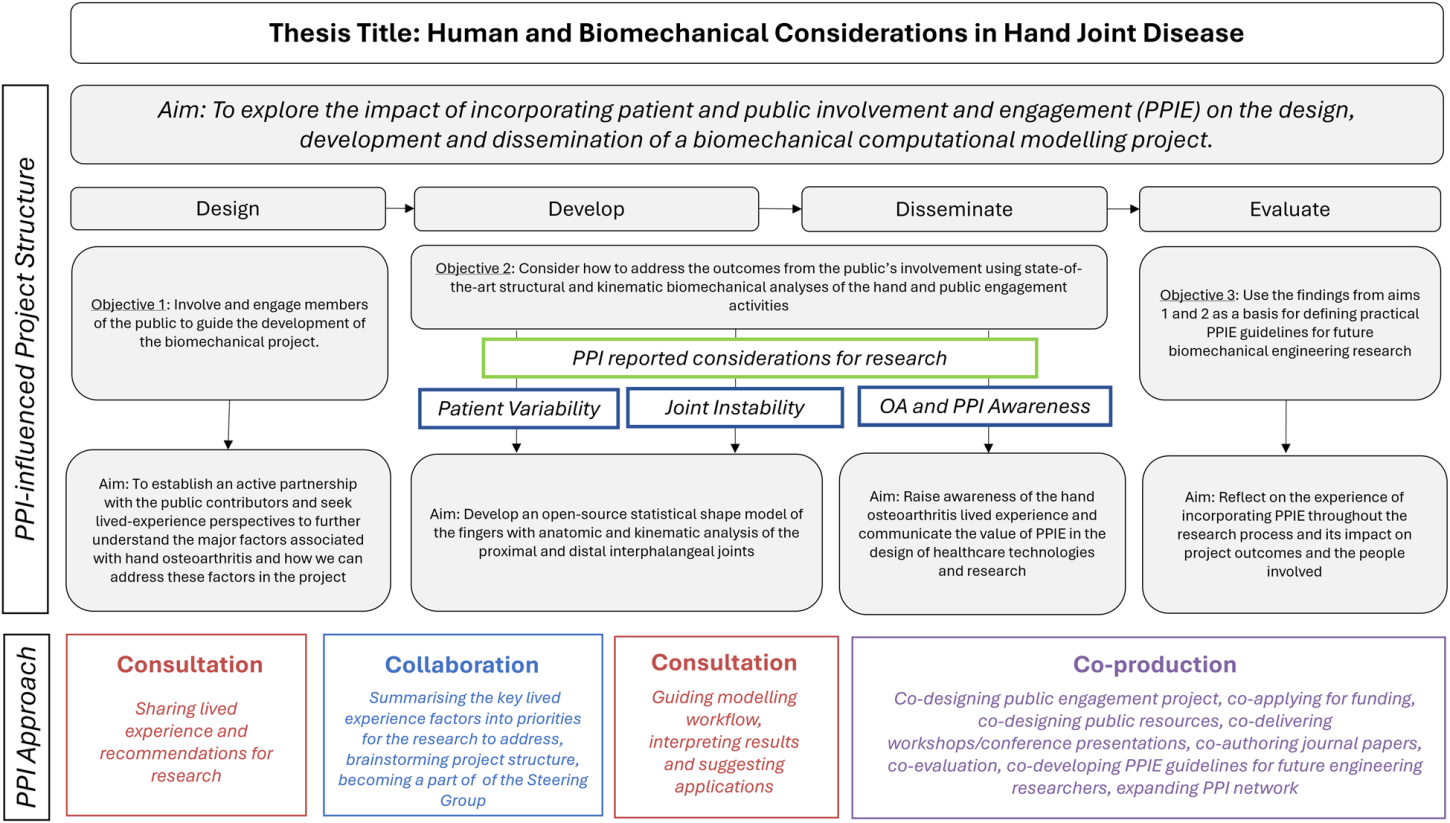
Final project plan (template inspired by Racine et al. [45]) detailing aims, objectives and methods influenced by PPI-identified priorities (patient variability, joint instability, OA and PPI awareness) and a summary of the public contributor role for each

This led to the diversification of our research outputs into two methodological groups:

- computational modelling and,
- public engagement,

to acknowledge three core PPI-identified research priorities. These included (1) patient variability, (2) joint instability and (3) hand OA awareness. This restructure also reprioritised the inclusion of the public contributors in the design, development, dissemination and evaluation of the project (Fig. 2). The PPI approach throughout the project alternated between consultative, collaborative and co-productive. The selected approach depended on the stage of research and contributors’ preferred level of involvement. An overview of the PPI approach, along with associated impacts and outcomes across all stages of the research project is presented in Table 2.

**Table 2:** Summary of PPI influence, impact and outcomes

Research Stage	Approach	Contributor role/influence	Impacts and Outcomes
Identifying the research problem	PPI group meetings	Highlighted the importance of acknowledging the public contributor insights in all stages of the project as well as at the beginning	Improved understanding of hand OA lived-experience Project aim and structure redefined Further validation of the scope for more active PPI in engineering Agreement to extend our partnership throughout the research process
Project design	PPI group meetings, group email discussions, review of early-stage discussion notes	Summarised key lived experience factors to facilitate project restructure Reviewed and agreed to suggested PPI activities within the study	Three core research priorities to drive the project workflow were identified. These included: patient variability, joint instability and raising hand OA awareness Contributors accepted invitation to sit on the project’s multidisciplinary steering committee
Commissioning: Internal funding for public engagement activity	PPI group meetings, steering group meetings, group email discussions, review and approval of funding applications via email	Became co-applicants on the funding call Reviewed and agreed on suggested PPI activities within the study	Development funding approved by Public Engagement with Research unit
Designing, managing and undertaking: Statistical Shape Modelling	PPI group meetings, group email discussions, steering group meetings, attending modelling and data collection demonstration	Highlighted the importance of considering how products or interventions may perform on different hand and finger shapes and sizes (i.e. “one size does not fit all,”) Feedback and interpretation given during model generation	Supported researcher’s critical thinking and approach to analysis: Guided data processing workflow (characterise the datasets before generating a dynamic model), purpose and usability (datasets publicly available with instructions of use—https://github.com/abel-research/OpenHands)
Designing, managing and undertaking: Kinematic Analysis	PPI group meetings, steering group meetings, attending modelling and data processing demonstrations	Highlighted joint instability as a major challenge associated with OA, likening it to a significant loss of function compared to what they would consider their ordinary level Feedback and interpretation given during workflow design	Supported researcher’s critical thinking and approach to analysis: The emphasis on function impacted the decision to combine the morphological data with associated kinematic data collected to further characterize the population in a joint instability context
Designing, managing and undertaking: Public Engagement	PPI group meetings, steering group meetings, public engagement project meetings	Highlighted a need to raise awareness of the hand OA lived experience within both the public and research community Attended meetings with student interns, guiding their work and enhancing their professional development	Sharing the contributor’s lived experience with OA via a dedicated website (https://handlethis.org/), social media campaign and accompanying infographic, delivery of PPI training material for engineering students, promotion of PPI within student, university-wide and community spaces and supporting student education and professional development
		Co-created and reviewed content for website and infographic Social media written content was reviewed to improve language for readability and accessibility Suggested events to attend and target audiences to engage	
Dissemination and implementation: Project outcomes	PPI group meetings, group email discussion, reviewing documentation	Co-designed and co-delivered dissemination material for target audiences Co-authored conference and journal papers, co-delivered training and co-presented at internal/external events	Dissemination of project outcomes and promotion of PPI within student, university-wide and community spaces, inspiring public members to get involved in research and encouraging researchers to create more PPI opportunities for public members
Evaluating impact	PPI group meetings, group email discussions	Offered reflections on overall PPI approach within the study	Recommendations for future work were devised based on views and feedback given in end-of-project reflective interviews

### PPI influence on computational modelling

Whilst the datasets do not represent an OA population, we highlight the potential of generating a transferable modelling methodology from an exemplar dataset, albeit free from hand or wrist disease or injury, that could be used to address population variability [46], predict risk of disease progression [38] and develop future pipelines for data more representative of the OA community.

Discussing data representativeness and model usability with our public contributors allowed our methodology to be scrutinised early on. Their influence guided the technical workflow, providing context to research efforts and supporting critical thinking. For instance, the prioritisation and generation of a statistical shape model to characterise the geometric variability with the data population came from the PPI discussions around patient variability. In addition, the emphasis on joint instability and it being likened to a “loss of function” by the public contributors also inspired correlation analysis between shape and kinematic data, further characterising the data population. This analysis revealed that if trained with additional CT images, the model may be of use for investigating further the apparent associations between joint conformity, which plays a role in stabilising the joint during movement, and movement quality, that may be descriptive of joint stability.

The resultant models created are first publicly available statistical shape models of the fingers’ skeletal anatomy generated from living participants ([47]). The finger models describe a small, homogeneous population, and assumptions cannot be made about how it represents individuals outside the training dataset however, the published model can supplement gross anthropometric datasets with additional shape information [48, 49], reaffirming its value for publishing open-source; another decision affirmed by contributors and academics alike.

### PPI influence on public engagement

Research questions are often formulated using published literature and although it frequently reports hand OA to be highly common [36, 37, 50]], [50–52] the language used can influence our awareness of its impact on an individual’s quality of life. For example, when discussing the burden of OA, Litwic et al. [53] state *that “even though the symptoms are often less disabling than when the knee or hip joints are involved, it can still significantly interfere with hand function.”.* In our PPI discussions, it was felt that this was an unfair statement since no one type of OA is more or less debilitating, they all manifest and impact quality of life in different ways. This type of comparison could lead to a gap in hand OA understanding, influencing people's decision to seek treatment or help. For instance, Dziedzic et al*.* [54] reported that whilst hand OA is common and has a significant impact and associated disability, many people living with the condition perceive that nothing can be done. Using semi-structured interviews, another study by Hill et al. [35] explored the experiences of adults aged 50 years and over, living with hand OA and reported that a key goal for them was to maintain independence; meaning they wanted to be self-reliant despite the limitations hand OA pose on daily living. In addition, they stated that hobbies and interests tended to be forgotten about to avoid the frustration associated with the inability to do them at the same pace or precision.

During the PPI consultations, we learnt a great deal about hand OA from each other and that helped recognise that public engagement efforts were needed to raise hand OA awareness within public and research communities. It has been judged unlikely that we would have got to this outcome without incorporating PPI; therefore, it was equally important for us to share our experiences of working together and in turn, increase PPI visibility  within engineering spaces as the project developed.

Alongside the development of the computational modelling efforts, public engagement content was co-produced with public contributors to engage various target groups. Designing and publishing a website dedicated to sharing the hand OA experience and attending a number of community events allowed us to both raise OA awareness and disseminate this project’s outputs. As the only known doctoral research project within the mechanical engineering department at the University of Southampton incorporating PPI in the research process, we hypothesised that this was not because other researchers did not want to, but because there is  limited  guidance or teaching on how to incorporate PPI in such a field. Therefore, as part of our public engagement efforts, we also co-created educational PPI material for the mechanical engineering undergraduate students at the university.

### Theory development

This research project transformed from a purely quantitative biomechanical analysis activity to a project influenced by the hand OA lived-experience, advocating for mutual learning with stakeholders often underrepresented (compared to clinicians and surgeons) in computational modelling work. Two of the three original contributors continued to be involved after the consultations up until the project’s completion. Therefore, though the number of contributors was small, we established meaningful and long-lasting partnerships. While the quality of the partnerships formed between researchers and the public are considered to significantly influence the effectiveness of PPI [13], we also acknowledge the enhanced robustness a larger and more diverse group could provide and encourage that for future studies.

Biomechanical engineering especially lends itself to a PPI-driven research approach since research often concerns a health condition that impacts a population. This further strengthens the relevance of the statement from Hewlett et al*.* [28] that patients “*have a personal experience of disease that is not available to most researchers, but that complements researchers’ analytical skills and scientific perspective*”. Our experience in this project has led us to strongly agree with this idea of complementary skills and its benefits to collaborative research, which is often highlighted as a requirement for effective multidisciplinary teams [55, 56].

Many practices in engineering design encompass the principles of PPI, such as the Design Council’s Double Diamond framework and human factors, and thus we do not believe our paper to be the first to delve into seeking non-engineer expertise. However we do specifically focus on the scope of public involvement in the development of engineering research. While there has been encouragement from bodies, such as the UK’s Engineering and Physical Sciences Research Council (EPSRC) [23, 24] and the Engineering Council [1], there are limited published examples of PPI in engineering research contexts. To avoid devaluing PPI, Ocloo and Matthews [57] suggest that researchers need to be trained and supported when undertaking public involvement. They state that by developing models of healthcare that are more co-designed and co-produced between all stakeholders, we can move beyond tokenism, share power, and create more equity in the decision-making process. This highlights the importance of increasing both the education and evidence-base of PPI in quantitative-based methodology as even if there has been a recent commitment to PPI from the engineering community, conceptual and practical barriers can still exist due to a lack of understanding from researchers regarding what it involves, how to support a diverse range of lay members, and the difference between PPI and qualitative research methods [58]. To support continued efforts to increase PPI in quantitative research, we need to continue contributing to the evidence base and share the lessons we have learned, including the successes and the challenges, from this approach.

### Impacts

This commentary presented the outcomes of early-stage consultations with members of the public living with hand OA and how they influenced the project's structure. Their involvement notably influenced and supported critical thinking, encouraging a new perspective and attitude toward the involvement of public members in engineering and quantitative methodological research. The overall impacts include:

#### Establishing and developing meaningful PPI relationships

- An active, ongoing and supportive partnership with two public contributors who were involved throughout the project.
- Frequent project updates and brainstorming meetings were organised with public contributors, particularly during the more co-productive stages of the project.
- Public contributors were invited to take and subsequently accepted roles of equal standing to supporting academics in the Project Steering Group
- Main changes/benefits for the doctoral researcher (TAM): A new perspective and attitude toward the involvement of public members in engineering/quantitative methodological research, driving the restructure of the project and advocacy for mutual learning with stakeholders often underrepresented (compared to clinicians and surgeons) in our work.
- Main changes/benefits for the public contributors (GL/NM): New opportunities to get involved in research relating to their lived-experience, contributing to research methods and attitude shifts to research within the department of mechanical engineering at the University of Southampton; working with researchers to encourage others to work with members of the public in similar ways.

#### Providing a case study for a PPI-driven biomechanical engineering project

- A project plan was designed to address considerations for researchers suggested by public contributors. These centred on:
  ai. Computational methods to assess bone joint shape and motion trends between patients to define levels of variability and joint instability.
  aii. Public engagement efforts to raise awareness of hand OA and the value of PPI including conducting workshops and developing digital and physical resources.
- Additional considerations were made to (1) address the representativeness and useability of computational modelling outcomes by making our work accessible to potential end-users and inviting collaborators to contribute to them and (2) engage with community groups and the biomedical engineering community, in particular early-stage engineers, to raise OA awareness and encourage active PPI practice.
- This project plan was continuously reviewed during Steering Group meetings (gathering of supervisory team, public contributors, students working in a similar field and stakeholders). A stakeholder map was created to outline the influence and interest of those involved or engaged during the project.

#### Creating a PPIE network

- We expanded our support network to include more individuals who advocate for this approach to engineering research and expanded our public network to include more voices (lived and non-lived experience) for current and future involvement and dissemination purposes.
- We created and delivered ‘PPIE in Engineering’ training material for early-stage biomedical engineering students to encourage more consideration of the public voice in their work. We also hosted opportunities for students to consult with us on best practice for incorporating PPI in their largely quantitative analysis-based projects.
- We established a consistent presence at local events to share our progress and invite members of the public to join the network.

### Recommendations for including PPI in quantitative-based research projects

These are not a ‘how to’ guide, instead an insight into the key takeaways from four years of experience of working with members of the public in a field that is largely driven by technology. These were written to summarise our key learnings and to encourage engineers in seeking research design input from the public.

#### The power of storytelling

*“For me, public involvement is important because you are getting all the information directly, not from books, journals, or other research but from the patients with OA pain of the hands.”*

*– Public contributor* [44]

Something that connects us all is that we each have a story to tell. Storytelling lends itself well to PPI and developing partnerships, because stories can inspire, empower, build empathy and educate, facilitating that element of PPI which encourages mutual learning [59]. Storytelling helps to establish connections between both people and ideas. We can learn facts from published literature or technical information from fellow researchers, but there are also things we can learn from the public such as perspectives and experiences that researchers often do not have and yet are often overlooked. PPI encourages focus on these areas and complements the resultant design, development and dissemination of the selected research methods. Listening and valuing the public’s expertise and the courage it takes to tells one’s story for the sake of research is crucial in developing purposeful research and accessible outputs.

*“Most scientists/engineers/academics/clinicians developing treatments for arthritis don’t have it. As a result, they can’t fully empathise, they can’t ‘feel’ what it’s like to live with arthritis – they need people with arthritis in the team to help all those involved in the project fully understand the condition.”*

*– Supporting Academic* [44]

#### Starting early

*“[The researchers] … who were running these sessions were quite surprised by some of the things that are affected by arthritis and what we have to do to overcome it. Simple things we take for granted like sewing, writing a letter, opening those childproof caps, or trying to. Even gripping a bread knife or picking something up, when we are in the throes of a flare-up can be nigh on impossible.”*

*– Public contributor * [44]

As a project timeline progresses and research methods have been established, people are more reluctant to make changes, so we suggest involving members of the public at the earliest stage possible. A question we were often asked throughout the project by engineers, was “How does PPI make a difference?” Here, we would invite people to look at PPI as something you *do* rather than something you measure. Listening to someone’s story can help to improve your understanding of a condition or a consensus of a research tool in an area you are working in, increasing the awareness of the societal impact of the research. Engineers often work together to develop research tools and thus, PPI can remove the barrier between them and the public, broadening the possible project outputs. PPI is evolutionary and thus difficult to predict how it will impact a project, therefore, leaving room to enact change by starting early is important.

#### Training/Planning

*“PPI group was not only interesting but very informative especially in the way that [the researcher] took notes and put them on the Jamboard. She was so very organised and her excellent technical skills with the computer meant that any points we made in our discussion were quickly put on the screen.”*

*– Public contributor* [44]

Barriers to implementing PPI in research are frequently attributed to a lack of understanding [57]. Planning is crucial for the smooth running of PPI activities and communicating with public contributors during a long-term project. Understanding the difference between involvement, engagement and participation is also important in helping to distinguish your intentions. While most useful when starting, we also stress the importance of seeking out guidance and reading up on relevant frameworks throughout the project. We commonly used: The GRIPP2 checklist [40], the UK Standards for Public Involvement [11], and the NIHR briefing notes [42] for PPI guidance and documentation from the National Co-ordinating Centre for Public Engagement (NCCPE) [60] for engagement guidance. We also advocate for learning on the job, therefore if there is a fellow researcher integrating PPI in their work, reach out to them for advice or an opportunity to shadow them to learn more about what it takes to involve members of the public in a research project and engage with different community groups.

#### Establishing partnership/project governance

*“These sessions weren’t about taking information from contributors; there was a mutual exchange of experience and mutual respect for everyone involved.”*

*– Postgraduate Researcher* [44]

*“Nothing about me, without me”*—the core principle of patient-centred care and decision-making embodied by various patient advocacy groups such as Patient Research Exchange and The Patient Association [61]—perfectly summarises the rationale behind establishing a working partnership with members of the public. Public contributors are members of the research team, steering the project and governing its outcomes. You are a team. A contributor’s perspective is equally valuable for shaping the purpose and outcomes of technical work and therefore, they should be consulted and treated with the same respect as a technical specialist or fellow researcher. How you value people’s input and time, plan meetings, communicate outside of meetings, and reward their efforts will contribute to the type of partnership cultivated. Avoiding tokenism, sharing power, and creating more equity in the decision-making process is easier when we move away from a transactional approach of working with the public to a reciprocal one. PPI can influence all stages of the research cycle; so when in doubt, ask the contributors *how* they would like to be involved and if their expectations are being fulfilled.

#### Commitment

*“[PPI] showed me that engineering continues to be technology-driven rather than patient-led. How can we expect people to use the technology we design if we haven’t considered their perspectives or needs in the design process? One way to do that is by involving them at every stage possible.”*

*– Postgraduate Researcher* [44]

Dawson et al*.* [13] argue that the success of PPI  is highly dependent on the relationships cultivated with the public contributors, in other words, one’s commitment to PPI. In an engineering context, the PPI commitment entails the researcher dividing their efforts between conducting the project on a technical-level to cultivating a partnership with public contributors and engaging with the different research communities. Viewing PPI as part of the research process and something that drives the project’s needs, rather than something extra to do, may help to map the required efforts and resources needed. As discussed Canney and Bielefeldt [7], this demonstrates the power of recognising the “*non-technical dimensions of engineering projects”* whereby skills including communication, budgeting, presenting to large audiences, managing expectations and note-taking compliment the process. These skills are just as important as the technical skills it may take to conduct the project as they ensure the smooth running of multiple different activities, facilitate transparency with all stakeholders and help provide a clear projection of the project’s expectations.

#### Flexibility

*“I hope that we can continue to work together and share our experience with the wider community because it has been great to see how our partnership has evolved since we first met and how many more opportunities we can create to share our work and communicate to researchers the value of public involvement.”*

*– Postgraduate Researcher* [44]

Making sure contributors can be involved at every possible stage is ideal but listening and respecting their preferred level of contribution for different activities is just as important. Working together in this way means the project must take a more holistic approach whereby the needs of all, including the researcher, the public contributors, supporting academics, the general public and the research community,  can be managed. To this end, it is important to diversify your communication approaches for each group. For instance, it’s important to ask the public whether the method that the project outputs have been presented resonates with them and reflects any previous contributions and if not, meetings can be organised to discuss alternative solutions. Managing expectations is reliant on strong communication, transparency and accountability. Being open, adapting to PPI outcomes and developing meaningful relationships with the public is incredibly beneficial as it may lead to new and unforeseen solutions or research outlooks. Lastly, it is important to see mistakes and misunderstandings as acceptable parts of the process. If you are honest with contributors, they will be honest with you. It may not be initially clear how to incorporate lived-experience insights into the project design but embracing the public-researcher partnership and being authentic and flexible will help. As discussed by Staley and Barron [62], learning is a PPI outcome.

#### Consistent feedback

*“Having input about our feelings towards arthritis and what we do to carry on normal life has felt like we are at last being listened to as well. I hope that more input from the patients will be taken into account as well in the future.”*

*– Public contributor* [44]

Feeding back to public contributors on acknowledgement, study progress, success and impact, is widely recognised as a common point of neglect by researchers conducting PPI [63, 64]. This feedback is crucial as it reinforces the concept of an equal partnership and eliminates the transactional nature of research. There will be stages  of the research process that the researcher undertakes independently, especially in an engineering context where data collection may include experimentation and testing of research material. It is important that you keep communication active by  recording  what was done and how PPI did or did not influence the work and provide contributors with this feedback even during the times where PPI is less embedded. After being involved , contributors appreciate evidence of their words/views being taken into account but also transparency of where it was not. This can also inform their contribution to future work. One way consistent feedback can be facilitated is by creating a steering group and inviting contributors to become members alongside supporting staff and stakeholders. Regular steering group meetings can play a crucial role in fostering a reciprocal relationship by providing a regular platform to keep everyone informed about the progress of the project and allow the opportunity for everyone to give feedback and suggest changes.

#### Diversification of efforts

The focus of quantitative-based projects revolves around data collection, processing and analysis. This project has shown how PPI encourages the clarification of the research question and prioritisation of research efforts with the consideration of an additional perspective, notably that of the lived-experience. Thus, PPI can help to determine the *purpose* of the technical investigations adopted; shifting our attitude from designing a project around the technology to using the technology as a means to address the needs of public contributors. This further emphasises how the technical (i.e. the computer programming, clinical or mechanical testing) and non-technical (i.e. developing working partnerships with the public or investing in interactive and accessible forms of steering a meeting) elements of a project can work side-by-side to define the research priorities, develop new ideas, acknowledge the public’s contributions, and at the very least make the research outputs more accessible.

### Conclusion

Developing a meaningful partnership between the public contributors allowed for the restructure of this doctoral research project, enabling a much heavier PPI influence than initially planned or published for a computational biomechanical engineering project. Involving members of the public can help generate ideas and guide engineering workflow; leading to solutions that may never have surfaced without collaboration. Although the evidence-base is limited, PPI has a place in quantitative-research driven fields such as engineering, especially biomedical engineering where people are often the end-users of research outcomes. In its simplest form, engineering is about using scientific knowledge to address people’s needs; how better to understand those needs than to consult, collaborate and co-produce with the public?

## Supplementary Information

Additional file 1.

Additional file 2.

## Data Availability

No datasets were generated or analysed during the current study.
