# Supplementary material for: “How would you handle this?” The impact of embedding early patient and public involvement in a biomechanical computational engineering doctoral research project
Source: Res Involv Engagem. 2025 Mar 18;11:26. doi: 10.1186/s40900-025-00694-3 (PMC11921647; doi:10.1186/s40900-025-00694-3)
Supplement: Supplementary file 1 — Additional file 1. [file 40900_2025_694_MOESM1_ESM.docx]

# **Terms of Reference – Hand osteoarthritis patient and public involvement consultations**

**Purpose/role of the group**

*Project summary*

Degeneration of hands and wrists presents a major socioeconomic problem and barrier to healthy ageing. In the UK, more than 1.5 million people have sought treatment for hand and wrist osteoarthritis (OA) which often affects joint movement and function; compromising the patient’s daily life [1]. Musculoskeletal modelling is a useful tool for investigating the key mechanisms of the hand, how these mechanisms are affected by disease and what the impacts of treatment are [2]. Tinashe Munyebvu has recently started a PhD at the University of Southampton and is investigating how the incorporation of the patient’s voice can influence the generation of musculoskeletal hand models capable of evaluating hand function and treatment options. This PhD project is in its early stages of development; therefore, using Patient and Public Involvement (PPI), this study aims to seek the involvement of members of the osteoarthritis community to help identify and prioritize the decisions made during the development of the musculoskeletal hand models.

*How much influence will people be able to have?*

This project is linked to, although not funded by, the EU project APRICOT. Therefore, it is possible that anonymized outputs from this project may be shared with APRICOT project partners. The EU Project, APRICOT aims to develop a radically new type of implant for the treatment of small joint arthritis. In this study, your influence will most likely be seen in the postgraduate research project rather than the larger APRICOT project. We hope to incorporate the experiences and voices of the members of the hand osteoarthritis community to inform the development and design of musculoskeletal hand models.

*Aims and Objectives*

- To enable members of the hand osteoarthritis community (the PPI contributors) to have an active partnership with researchers.
- To discuss and outline the major issues associated with hand arthritis.
- To explore the challenges associated with current hand arthritis treatments.
- To discuss key areas of investigation that can be facilitated by computer-generated hand models (i.e., hand function, treatment evaluation etc.)

**Membership**

1. Members:
   - Tinashe Munyebvu (Postgraduate Researcher, Faculty of Engineering and Physical Sciences)
   - Dr Charles Burson-Thomas (Research Fellow, Faculty of Engineering and Physical Sciences)
   - PPI contributors (members of the hand osteoarthritis community)
2. PPI contributors: Public contributors will receive expenses and reimbursement in line with the NIHR Southampton BRC payment policy for all activities relating to these consultations
3. Quorum: At least half of the group members should be present for a meeting to proceed otherwise that session will be rescheduled.

Tinashe Munyebvu will send invites to members of the group and will be aided by Charlie Burson-Thomas. Guests are welcome to join by invitation from members of the group as and when the agenda dictates.

**Sessions**

We are hoping to conduct a total of four PPI sessions (one every two weeks). Each session will last no more than an hour and will be held online via video-conferencing software (Microsoft Teams).

*What is expected from members of the public?*

Provide an honest description of the challenges of living with hand osteoarthritis, based on personal experience. Contributors should be reassured that there is no ‘right answer’: we would like to listen to your experience and learn with curiosity. Also, we would expect that should two people have a different experience, they would not be questioned by each other regarding the validity of their descriptions – each participant's views will be respected by all.

*What can participants expect from us?*

Members of the public can expect us to be polite and honest, show proper consideration of their views, and show respect regarding their knowledge and experiences. We will also ensure we are sincere when describing the views of the people involved – we will never deliberately misrepresent anyone’s views.

*Anonymity, recording and sharing information*

It is important that members can speak freely of their views. Sessions will not and should not be recorded using video/audio. Points discussed verbally during PPI sessions will be recorded by one of the investigators in the form of hand-written notes. These notes act as a written record of a meeting (minutes). They will be transcribed into digital notes after each session and sent via email to every present member of the corresponding meeting. When recording information or discussing any of the information publicly, there will be no attribution of comments or views of an individual. At any point during or after the PPI sessions, the PPI contributors will have the right to access, correct, and/or delete recorded information they feel does not represent what was discussed.

Personal data such as names and emails will not be shared with anyone outside the research team (Tinashe, Charlie and the supervisory team).

*Group responsibilities*

- An invite link for the Microsoft Teams meeting will be sent no later than two working days before the meeting (please do not share this link with anyone outside of the group)
- Group members are encouraged to inform Tinashe Munyebvu of any topics they would like raised before the next meeting
- Within the first ten minutes of the meeting, Tinashe Munyebvu will provide a summary of the project’s progress including a summary of the previous meeting
- Following the project summary, the rest of the meeting will consist of talking around each point on the agenda
- Minutes of each meeting will be circulated and agreed by all those who attended the meeting
- Members may be contacted between sessions - contact between group members can be done via email unless agreed otherwise.

**Review**

The information in this terms of reference will be reviewed biweekly to ensure it is in line with current practice.

**References**

[1] Arthritis Research UK (2013), “OSTEOARTHRITIS IN GENERAL PRACTICE Data and perspectives,” 2013.

[2] M. Mirakhorlo, J. M. A. Visser, B. A. A. X. Goislard de Monsabert, F. C. T. van der Helm, H. Maas, and H. E. J. Veeger, “Anatomical parameters for musculoskeletal modeling of the hand and wrist,” Int. Biomech., vol. 3, no. 1, pp. 40–49, Jan. 2016, doi: 10.1080/23335432.2016.1191373.
