# Supplementary material for: “How would you handle this?” The impact of embedding early patient and public involvement in a biomechanical computational engineering doctoral research project
Source: Res Involv Engagem. 2025 Mar 18;11:26. doi: 10.1186/s40900-025-00694-3 (PMC11921647; doi:10.1186/s40900-025-00694-3)
Supplement: Supplementary file 2 — Additional file 2. [file 40900_2025_694_MOESM2_ESM.docx]

**Supporting File 2 - Impact log for PPI consultations during the planning/design phase of the doctoral research project (Feb/Mar 2021)**

| **Date** | **Outcome*** | **Impact**** | **Reflections/learning** |
| --- | --- | --- | --- |
| *03/02/2021* | *First session: introductions and insight with everyone’s connections and why they wanted to get involved in this project. Public contributors were given a brief presentation on the PhD project and its current status. We got into a brief discussion about pain relief, age representation in OA research and the impact of joint instability of daily living. Meeting minutes were sent to everyone who took part for review.* | *As the first session, we prioritised getting to know each other and outlining the project and plan for their involvement. Though we didn’t get into much discussion about the lived-experience, it was clear that everyone was keen to get involved and share their stories.* | *Public contributors were involved in previous PPI sessions; however this was pre-COVID19 pandemic/lockdown/social distancing. As these sessions took place during a lockdown, we were all adapting to this new way of engaging with people. We tried to use a university-favoured video conferencing software to host the call however it wasn’t very compatible to those without university accounts. Therefore, we will use a more public-friendly application.* |
| *17/02/2021* | *The lived-experience discussions began we talked the sorts of activities that involve hands, and which are important and/or impacted by hand OA, the usefulness of a computer model of the human hand (the backbone of the PhD), clinical consideration and age representation of hand OA.* | *Aspects of the hand OA lived-experience were discussed; including nutritional or weather factors that were unbeknownst to the researchers. Considerations for the computational modelling aspect of the project were discussed and noted. These included the applications (i.e. education, research, rehabilitation, surgery) for such a model and who would benefit the most from its outcomes. In addition, the data used for such computer models was discussed (not one size fits all). This was key consideration for the computational modelling. An invitation to the project Steering Group was extended to the public contributors.* | *We got into interesting conversations however since TM also had to record the minutes, the conversation could get affected at times when a pause was needed to write things down. Therefore, a more engaging way of recording the outcomes of the meetings was needed. In addition, we were all still getting to know each other so a more interactive way of capturing the discussion would be welcome.* |
| *03/03/2021* | *Continued the conversation of activities of daily living (ADLs) impacted by hand OA. These were listed and captured on the interactive whiteboard. We took the ADLs and tried to categorise the types of movements needed for them. For example, the ADLs that appeared most affected by hand OA were those that required small and repetitive. We talked about self-management techniques (adaptation). We also discussed was joint instability. This session also focused on views on treatment and considerations for new implants. We discussed the conditions of OA joints and things that public contributors would need to take into consideration before signing up for an implant surgery. This included trust and transparency with the clinical team, anaesthetic preferences and preserving as much joint tissue as possible* | *There was an agreement that public awareness of hand OA appeared lower than for hip or knee OA. Public contributors thought it was important for the PhD to find ways to raise more awareness. Joint instability has frequently come up during discussion with the public (pilot study and this work). Pain and stiffness are usually highlighted in the literature as a major problem of OA rather than joint instability. Joint instability has a major impact of hand function and “lack of trust” in joints as mentioned by the public contributors. Again, this might be something we can address with the project. The last part of the session mostly focused on treatment considerations. This linked closely with the outcomes of the APRICOT project rather that the PhD however, their views and opinions on clinical translation of such a device sparked conversations of their involvement in the research process and what they would consider important before seeking out such treatment* | *The incorporation of Google Jamboard stimulated a much more active discussion. We covered a lot of topics, especially on the lived-experience, and there was a lot that we agreed needed more awareness both within in the public and research community. This was the first instance that we wondered if we could do that together, rather than isolating the PPI to these four project design consultations.* |
| *17/03/2021* | *We aimed to cover more on the treatment. We discussed their experience with diagnosis, treatment and rehabilitation. This led to more considerations on how researchers and clinicians could improve from a patient perspective. This led to another discussion on general awareness of hand OA and the lack of information in comparison to the hips and knees. We used the last few minutes of the session for APRICOT related discussions, this included the benefits of early inventions and a multiple choice question to outline a preferred scenario for the “perfect” implant to treat hand OA.* | *As we discussed everyone’s views on the clinical and surgical pathways for treatment, we got a better understanding of the needs public contributors wish to be addressed or improved. This paired with the need for more public information on hand OA once again highlighted their enthusiasm to share their experiences with the public and get the conversation started about hand OA.* | *TM used a blank interactive whiteboard the session before. The use of a custom whiteboard template this time around helped with reformatting after the meeting. It was now just a case of putting the post-it’s in the right area. Our work together is only beginning. We are all fairly new to PPI, but it was clear that there was much more we could talk about and learn from each other; four meetings was not enough. We plan to schedule more.* |

Attendees to all sessions: TM (PhD Student), CBT (Academic Support), CP (Public contributor), GL (Public contributor) , NM (Public contributor)

*Outcome: Immediate, demonstrable change

**Impact: Sustained change, can be intended/unintended, positive/negative
